# Incidence, casualties and risk characteristics of civilian explosion blast injury in China: 2000—2017 data from the state Administration of Work Safety

**DOI:** 10.1186/s40779-020-00257-5

**Published:** 2020-06-11

**Authors:** Xu Wang, Juan Du, Zhuo Zhuang, Zheng-Guo Wang, Jian-Xin Jiang, Ce Yang

**Affiliations:** 1 State Key Laboratory of Trauma, Burn and Combined Injury, Research Institute of Surgery, Daping Hospital, Army Medical University, Chongqing, 400042 China; 2 Department of Emergency, the Second Affiliated Hospital of Medical College in Zhejiang University, Hangzhou, 310009 China; 3 Applied Mechanics Laboratory, School of Aerospace, Tsinghua University, Beijing, 100084 China

**Keywords:** Explosion, Blast exposure, Blast mitigation, Overpressure, trauma and injury

## Abstract

**Background:**

Civilian explosion blast injury is more frequent in developing countries, including China. However, the incidence, casualties, and characteristics of such incidents in China are unknown.

**Methods:**

This is a retrospective analysis of the State Administration of Work Safety database. Incidents during a period from January 1, 2000 to April 30, 2017 were included in the analysis. The explosions were classified based on the number of deaths into extraordinarily major, major, serious and ordinary type. Descriptive statistics was used to analyze the incidence and characteristics of the explosions. Correlation analysis was performed to examine the potential correlations among various variables.

**Results:**

Data base search identified a total of 2098 explosions from 2000 to 2017, with 29,579 casualties: 15,788 deaths (53.4%), 12,637 injured (42.7%) and 1154 missing (3.9%). Majority of the explosions were serious type (65.4%). The number of deaths (39.5%) was also highest with the serious type (*P* = 0.006). The highest incidence was observed in the fourth quarter of the year (October to December), and at 9:00–11:00 am and 4:00–6:00 pm of the day. The explosions were most frequent in coal-producing provinces (Guizhou and Shanxi Province). Coal mine gas explosions resulted majority of the deaths (9620, 60.9%). The number of explosion accidents closely correlated with economic output (regional economy and national GDP growth rate) (*r* = − 0.372, *P* = 0.040; *r* = 0.629, *P* = 0.028).

**Conclusions:**

The incidence and civilian casualties due to explosions remain unacceptabe in developing China. Measures that mitigate the risk factors are of urgently required.

## Background

The various risks in daily production determine the long-term likelihood of explosives accidents [1, 2]. In particular, recent giant explosives accidents (e.g., “8.12” giant explosion in Tianjin harbor and “8.2” Kunshan explosion in Jiangsu) emphasized the urgent need for prompt action [3, 4]. A major obstacle in designing and implementing effective measures to curb such incidents and emergency responses is the lack of basic information, including the incidence, the type, and risk factors [5, 6].

In civilian explosions, blast injuries are generally categorized as primary, secondary, tertiary and quaternary types [7, 8]. Civilian explosive blasts often occur in closed or semi-closed spaces. Primary blast injuries may be amplified by wave reflection and superposition from the surrounding walls rather [2, 5, 7, 8]. Tympanic membranes, lungs, intestinal ducts and the brain are the most frequently injured organs among survivors [9–14]. Secondary blast injuries are caused by flying debris [15, 16]. Tertiary blast injuries occur when a victim is physically displaced by the force of air movement or is crushed by structural collapse, often in dilapidated buildings such as factories, folk houses and underground shafts [3, 17]. Quaternary blast injuries mainly include burns and inhalational injury, and are often seen in explosions caused by chemical compounds or petroleum [2, 3, 18]. In compared to soldiers in combat, civilians often lack training and personal protective equipment, and thus are prone to more severe injuries [19–21].

Existing literature recognizes the influence of economic development on traffic accidents [22, 23]. However, whether civilian explosives accidents are associated with economic development is unknown. In the present study aims, we conducted a retrospective analysis of the State Administration of Work Safety (SAWS) database, and systematically examined the profiles of civilian explosive blast injuries after 2000.

## Methods

### Data collection

The State Administration of Work Safety (SAWS) is a nation-wide database of civilian casualties caused by explosions (http://www.chinasafety.gov.cn/newpage) in China. Reporting to SAWS is mandatory. The SAWS database was searched for casualty records of all casualties caused by explosions between January 1, 2000 and April 30, 2017 using “explosions”, “low speed detonation” and “deflagration” as the search terms. Terrorism-related events were not included. Reports published by government-sponsored websites (Xinhua News Agency, China News Service, China Daily, People’s Daily) were reviewed to verify the accuracy and completeness of the data retrieved from the SAWS database. We also searched PubMed, Web of Science and ProQuest databases for possible missing accidents using the search terms “explosive”, “explosion”, “blast” and “overpressure” to further verify the integrity of the data from the SAWS database.

National population, gross domestic product (GDP) and GDP per capita were collected from the National Bureau of Statistics of the People’s Republic of China. This manuscript was drafted in compliance with the Strengthening the Reporting of Observational Studies in Epidemiology (STROBE) statement.

### Ethical approval

This study was approved by the Institutional Review Board and the Medical Ethics Committee of Daping Hospital, the Army Medical University, China (HUUWEC2017017).

### Data analysis

Annual incidence of explosion casualties, including death, injured, injury severity and missing, was calculated. The trend was analyzed by correlation analysis based on incident year. The explosion was graded based on the number of deaths into: ordinary (*n* < 3), serious (3 ≤ *n* < 10), major (10 ≤ *n* < 30) or extraordinarily major (*n* > 30). To determine whether the explosion blast injury follow a temporal pattern, a Pearson correlation analysis was performed between the numbers of explosions and the timing (quarter, month, day and time).

### Statistical analysis

All statistical analyses were conducted using SPSS 24.0 for Windows (Shareware 1050, IBM, USA). Categorical variables, presented as numbers and percentage, were analyzed with Chi-Square tests. Correlation analysis was performed to examine the potential correlations among continuous variables. Descriptive statistical methods were used to analyze the grade and type of explosion, explosion charges, and geographical location. *P* < 0.05 (2-sided) were considered significant.

## Results

### Incidence of explosions and casualties

**Fig. 1:**
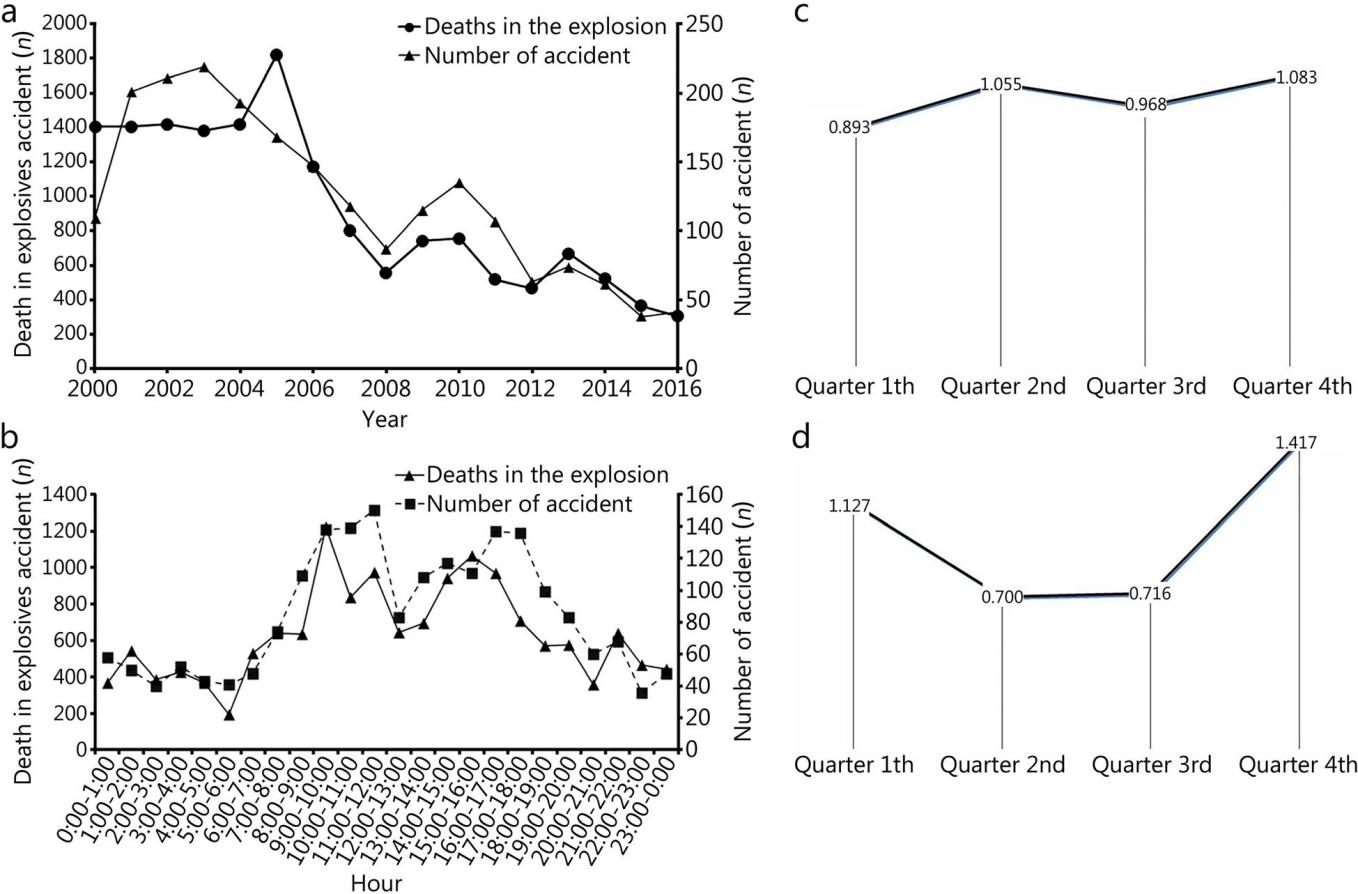
Correlation of deaths and number of explosives in China from 2000 to 2017. **a**: Correlation of deaths and number of explosives accidents by year; **b**: Correlation of deaths and timing of the explosion during the day; **c**: Seasonal pattern; **d**: Season pattern of coal mine explosions

A total of 2098 explosion accidents were identified in the SAWS database during a period from January 1, 2000 and April 30, 2017. The total casualty was 29,579: 15,788 deaths (53.4%), 6130 severe injuries (20.7%), 6507 minor injuries (22.0%), and 1154 missing (3.9%). Pearson correlation analysis revealed a positive correlation between the total number of explosion accidents with the number of deaths (*r* = 0.881, *P* = 0.0004, Table 1, Fig. 1a). The 2004/2005 years had the highest number of accidents and deaths. There was a general tendency for decreasing incidence of explosion blast injury as well as death per million people after 2001.

**Table 1:** Incidence and casualties by year

Year	Total accidents (*n* = 2098)[*n*(%)]	Total casualty (*n* = 29,579) [*n*(%)]	Injured (*n* = 12,637) [*n*(%)]	Deaths (*n* = 15,788) [*n*(%)]	Missing (*n* = 1154) [*n*(%)]	Total population(/10^7^)	Incidence of blast injury per million people^*^	Death rate per million people^#^
2017^**a**^	11(0.5)	86(0.3)	35(0.3)	48(0.3)	3(0.3)	_	_	_
2016	41(2.0)	576(1.9)	260(2.1)	306(1.9)	10(0.9)	139.0	0.29	2.20
2015	38(1.8)	1878(6.3)	1502(11.9)	362(2.3)	14(1.2)	137.5	0.28	2.63
2014	61(2.9)	1443(4.9)	915(7.2)	523(3.3)	5(0.4)	136.8	0.45	3.82
2013	74(3.5)	1490(5.0)	805(6.4)	671(4.3)	14(1.2)	136.1	0.54	4.93
2012	63(3.0)	1019(3.4)	539(4.3)	467(3.0)	13(1.1)	135.4	0.47	3.45
2011	107(5.1)	1241(4.2)	686(5.4)	515(3.3)	40(3.5)	134.8	0.79	3.82
2010	135(6.4)	1981 (6.7)	1182(9.4)	756(4.8)	43(3.7)	134.1	1.01	5.64
2009	115(5.5)	1480(5.0)	700(5.5)	744(4.7)	36(3.1)	133.5	0.86	5.57
2008	87(4.1)	1049(3.5)	464(3.7)	559(3.5)	26(2.3)	132.8	0.66	4.21
2007	118(5.6)	1381(4.7)	507(4.0)	804(5.1)	70(6.1)	132.1	0.89	6.09
2006	147(7.0)	1979(6.7)	747(5.9)	1173(7.4)	59(5.1)	131.5	1.12	8.92
2005	168(8.0)	2701(9.1)	814(6.4)	1824(11.6)	63(5.5)	130.8	1.28	13.94
2004	193(9.2)	2275(7.7)	741(5.9)	1421(9.0)	113(9.8)	130.0	1.48	10.93
2003	219(10.4)	2390(8.1)	906(7.2)	1382(8.8)	102(8.8)	129.2	1.70	10.70
2002	211(10.1)	2051(6.9)	512(4.1)	1421(9.0)	118(10.2)	128.5	1.64	11.06
2001	201(9.6)	2171(7.3)	535(4.2)	1407(8.9)	229(19.8)	127.6	1.58	11.03
2000	109(5.2)	2388(8.1)	787 (6.2)	1405(8.9)	196(17.0)	126.7	0.86	11.09

^*^ The incidence of blast injury in millions of people = Number of accidents / Total population × 10^6^; ^#^ Death rate of millions of people = Deaths / Total population of China × 10^6^; ^a^Only the first four months were included in 2017; "-". No data

### Explosion grade vs. casualties

Among the 2098 explosions, the number of accidents was 79 for extraordinarily major accident (3.8%), 283 for major accident (13.5%), 1372 for serious accident (65.4%) and 364 for ordinary accident (17.3%), with the largest number in the serious category (*P* = 0.03). The mean number of casualties and deaths in the extraordinarily major accidents was 104.3 and 58.0 per accident, respectively, both highest among the four categories. There was no statistical difference between the number of severe and minor injuries. The number of missing was 3.8% of the total casualties, with the highest number in the ordinary category (S Table 1).

### Timing of the explosions

The fourth quarter of the year (October – December) had the highest incidence of explosion accidents. With the day, the explosions were most frequent at 9:00–11:00 am and 4:00–6:00 pm. Pearson correlation analysis showed a correlation between the number of accidents with the number of deaths by time interval (*r* = 0.857, *P* = 0.006, Fig. 1b). No temporal pattern was identified with regards to the day within a month or month within a year (Fig. 1c).

### Geographical distribution

**Fig. 2:**
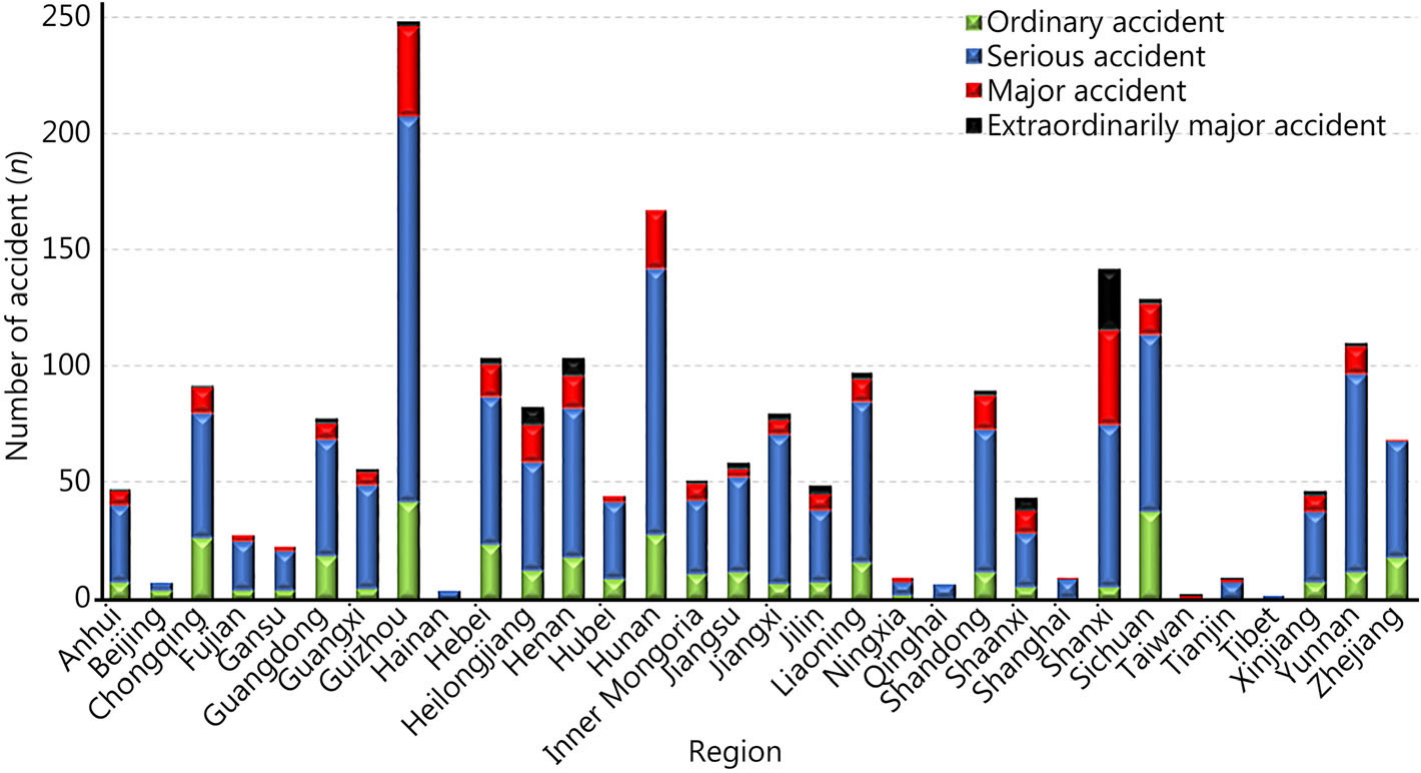
Analysis of different grades of explosion accidents by region. Grade of explosion was categorized by number of deaths into ordinary (*n* < 3), serious (3 ≤ *n* < 10), major (10 ≤ *n* < 30) or extraordinarily major (*n* > 30)

The highest incidence and deaths were identified in Guizhou Province in southwest China (248, 11.8%) and Shanxi Province in northern China (2289, 14.6%), respectively. Extraordinarily major accidents were most common in Shanxi (26, 32.9%), Henan (8, 10.1%) and Heilongjiang (8, 10.1%). Major accidents were also common in Shanxi (41, 14.5%), Guizhou (39, 13.8%) and Hunan (25, 8.8%). Serious accidents were most frequent in Guizhou (165, 12.0%) and Hunan (114, 8.3%). Ordinary accidents were most common in Guizhou (42, 11.5%) and Sichuan (38, 10.4%) (Fig. 2**)**. Pearson correlation analysis revealed a positive correlation between the number of accidents with the number of deaths by regions (*r* = 0.827, *P* = 0.002).

### Causes of explosions and relationship with casualties

The causes of explosions were categorized into seven types: coal mine gas, fireworks, blasting agent, chemical product, high-pressure equipment, flammable explosives and others. More than three quarters of the explosions resulted from coal mine gas (1029, 49.1%), fireworks (323, 15.4%) or flammable explosives (260, 12.4%, Table 2).

**Table 2:** Casualty breakdown by explosion types [*n*(%)]

Explosive types	Number of accidents	Total casualty	Patients with severe injuries	Patients with minor injuries	Deaths	Missing
Coal mine gas	1029 (49.0)	14,360(48.5)	1641(26.8)	2191(33.7)	9620(60.9)	908(78.7)
Fireworks	323 (15.4)	3945(13.3)	907(14.8)	1105(17.0)	1857(11.8)	76(6.6)
Explosive blasting agent	187 (8.9)	2970(10.0)	683(11.1)	937(14.4)	1286(8.1)	64(5.5)
Chemical product	158 (7.5)	2897(9.8)	583(9.5)	1280(19.7)	992(6.3)	42(3.6)
High pressure equipment	112 (5.3)	919(3.1)	333(5.4)	130(2.0)	449(2.8)	7(0.6)
Flammable explosive	260 (12.4)	4260(14.4)	1921(31.3)	816(12.5)	1468(9.3)	55(4.8)
Other	29 (1.4)	228(0.8)	62(1.0)	48(0.7)	116(0.7)	2(0.2)

Among the various causes of explosions, coal mine gas explosions resulted in the highest casualties (14,360, 48.6%): 9620 deaths (67.0%) and 3832 injured (26.7%). The percentage of severe injuries in explosions due to flammable explosives and high-pressure equipment was 45.1% (1921) and 36.2% (333), respectively. Furthermore, the explosion of coal mine gas (212, 85.0%) was mostly seen among these regions. Coal mine gas explosions occurred more frequently in the second quarter of the year (April, May and June). Explosions due to fireworks were more frequent in the first and fourth quarters, and particularly in January and November (S Table 2, Fig. 1d).

### Explosion accidents vs. economic development

**Fig. 3:**
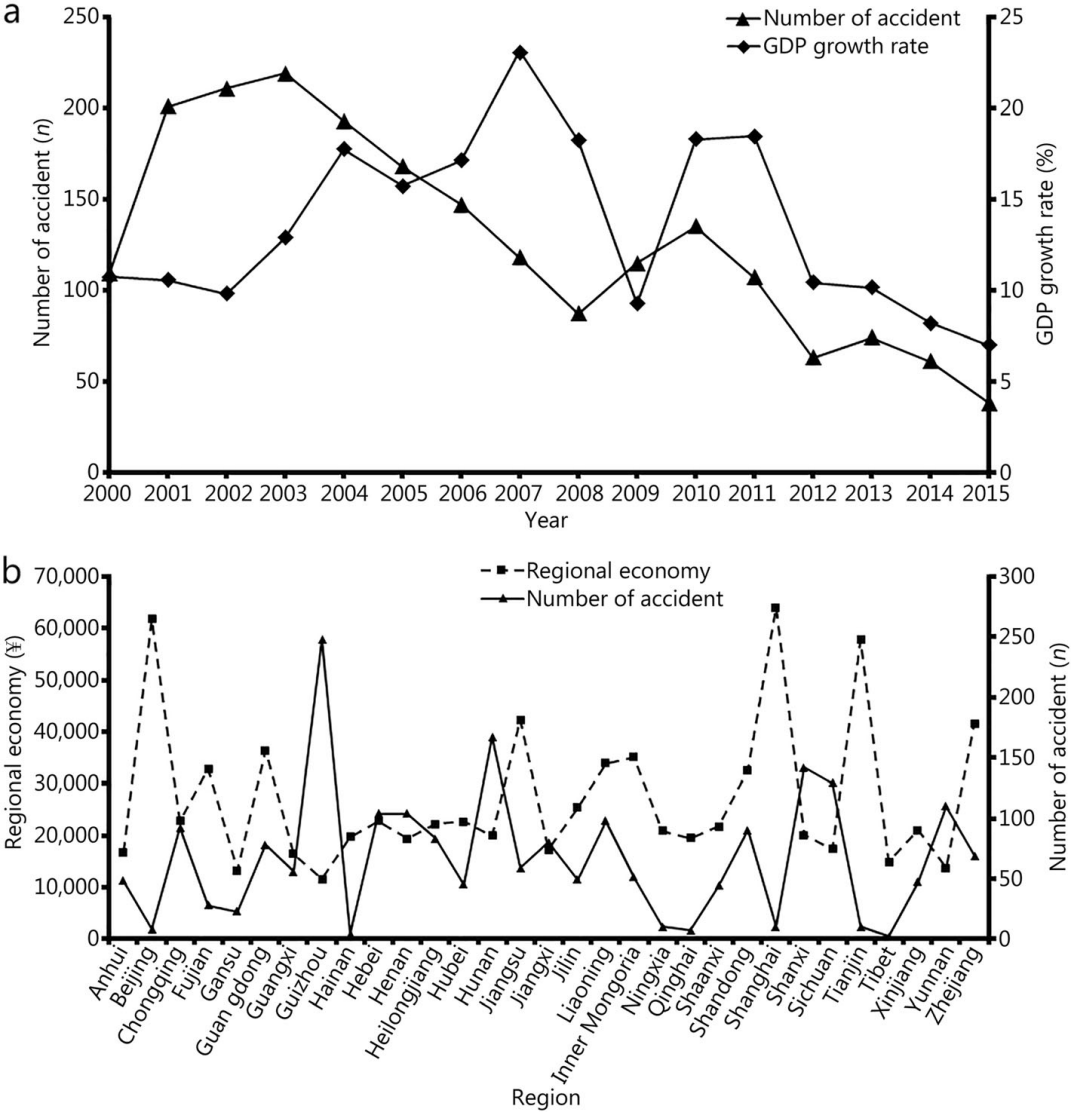
Correlation of explosion accidents and economic development. **a**: Correlation with GDP growth rate; **b**: Correlation with regional economy

Pearson correlation analysis indicated a correlation between the regional population and economy (regional economy and national GDP growth rate) with the number of accidents (*r* = 0.470, *P* = 0.008; *r* = − 0.372, *P* = 0.040; *r* = 0.629, *P* = 0.028, Fig. 3, S Table 2).

### Explosions and potential protection

Coal mine gas explosions were the most frequent cause of the explosion accidents (126, 24.5%). Seventy point 6 % (363 cases) of the explosion charges were coal mine gas, pyrotechnics, ignitable gas and explosive chemical compound among the 11 material subtypes (Table 3).

**Table 3:** The number of explosions by explosion charges

Material subtype	2010	2011	2012	2013	2014	2015	2016	Total [*n*(%)]
Coal mine gas	33	28	14	20	14	6	11	126(24.5)
Pyrotechnics	30	22	7	16	6	5	7	93(18.1)
Ignitable gas	16	12	14	9	14	11	2	78(15.2)
Explosive chemical compound	15	16	6	7	7	8	7	66(12.8)
Other sealed equipment	6	7	11	7	2	1	2	36(7.0)
Explosive blasting agent	12	5	5	6	6	0	1	35(6.8)
Ignitable liquid	10	2	1	4	6	1	1	25(4.9)
Ignitable solid	5	5	1	1	3	2	4	21(4.1)
Boiler	3	5	3	1	0	0	4	16(3.1)
Other (Not identified)	2	4	0	2	2	0	2	12(2.3)
Black powder/black powder Substitutes	1	0	0	0	1	4	0	6(1.2)

## Discussion

The current study identified a total of 2098 explosions, with 29,579 casualties during a period from January 1, 2000 and April 30, 2017. On average, one explosion accident happened every 2.8 days; nearly five people suffered from blast injury every day. The mean number of casualties per explosion accident was fourteen. The mean number of deaths in the extraordinarily major accidents was 58.0 per accident.

In general, civilians are poorly prepared to handle the severe emotional, logistical and medical burden of a sudden, large influx of casualties [24]. Upon a catastrophe, medical facilities must be prepared to manage massive numbers of severely injured patients [25]. The current study indicated a decline in morbidity and mortality in civilian explosion accidents from 2000 to 2017 in China, but extraordinarily major accidents remain a dire challenge for the emergency rescue teams. Large number of patients must be triaged and treated in the chaotic circumstances within a short period of time. Decreasing number of in missing victims during the study period in the current study may be attributed to improvements in search strategies and equipment as well as the increasing use of DNA identification [26].

Explosives accidents tended to occur at 9:00–11:00 am or 4:00–6:00 pm during the day. These times generally relate to busy activity by majority of the facility personnel. The fact that explosion accidents were most frequent in the fourth quarter of every year is consistent with the high volume of business production towards the end of year. The use of heating systems might also have contributed to the high incidence in the fourth quarter of the year. Thus, we recommend that early warning systems be installed at these specific time intervals to alert for the high risk of explosions.

Explosion accidents and mass casualties are most frequent in coal-producing provinces. Resolving this issue clearly depends on alternative energy sources other than coal [27–29]. Since nearly half of explosives accidents resulted from coal mine gas, most of the victims in these coal mine explosions sustain serious blast injuries [30].

As a developing country, the principal risk factors in China of explosion accidents remain industrial causes. The current study suggested that, in addition to advanced search-and-rescue systems, emphasis must be placed in addressing un-planned expansion of industrial production [31, 32]. At the level of individualized protection, coal mine-related personnel should be provided with equipment that absorb blast waves [33–35] in addition to bulletproof materials [36] to minimize blast injury.

There are several limitations in the current study. First, we do not know the extent of non-reporting in the SAWS database despite of its mandatory nature. The second limitation is the lack of information on long-term physical, psychological and social impact of the explosions on survivors. It is also necessary to analyze the causes of death, injury types and emergency rescue and medical response effort in more details in future studies.

## Conclusions

In summary, civilian explosion is unacceptably common, with high casualties in China despite of a decline in the past few years. The overall strategy of injury relief is effective, but more individualized and universal protective measures are needed.

## Supplementary information

**Additional file 1: Table S1.** Casualty by explosion accidents grade [*n*(%)].

**Additional file 2: Table S2.** Correlation analysis of explosion accidents and regional population and economy.

## Data Availability

All authors had full access to all the data in the study.

## Abbreviations

GDP: Gross domestic product
SAWS: State Administration of Work Safety
STROBE: The Strengthening the Reporting of Observational Studies in Epidemiology
