# Supplementary material for: Incidence, casualties and risk characteristics of civilian explosion blast injury in China: 2000—2017 data from the state Administration of Work Safety
Source: Mil Med Res. 2020 Jun 11;7:29. doi: 10.1186/s40779-020-00257-5 (PMC7288536; doi:10.1186/s40779-020-00257-5)
Supplement: Supplementary file 1 — Additional file 1: Table S1. Casualty by explosion accidents grade [n(%)]. [file 40779_2020_257_MOESM1_ESM.docx]

**Supplement Table 1** Casualty by explosion accidents grade [*n*(%)]

| Accident grade* | Total accidents (*n*=2098) | Severe injuries (*n* =6130) | Minor injuries (*n* =6507) | Deaths (*n*=15,788) | Missing (*n* =1154) | Casualties per accident |
| --- | --- | --- | --- | --- | --- | --- |
| Extraordinarily major accident | 79(3.8) | 1175(19.2) | 2484(38.2) | 4583(29.0) | 44(3.8) | 104.3(72.8) |
| [Major accident](http://www.baidu.com/link?url=ZZ7yVS050vhX7Z4yNS3UDO1EyuyfmQDdNATSB-daQJLtlLi5HAm4IMnqqCM_4S_iKWXOq10d-QWJVyjR-RxJr8PC5E_3Q8Mw_-tgJ4QXdhjYvygAEpcDENJyKLKMvmGb) | 283(13.5) | 1527(24.9) | 1741(26.8) | 4519(28.6) | 201(17.4) | 27.5(19.2) |
| Serious accident | 1372(65.4) | 2937(47.9) | 1988(30.6) | 6236(39.5) | 369(32.0) | 8.1(5.7) |
| Ordinary accident | 364(17.3) | 491(8.0) | 294(4.5) | 450(2.9) | 540(46.8) | 3.4(2.4) |

*****Grade of explosion was categorized based on the number of deaths into ordinary (*n* < 3), serious (3 ≤ *n* < 10), major (10 ≤ *n* < 30) or extraordinarily major (*n* > 30).
