# Supplementary material for: Incidence, casualties and risk characteristics of civilian explosion blast injury in China: 2000—2017 data from the state Administration of Work Safety
Source: Mil Med Res. 2020 Jun 11;7:29. doi: 10.1186/s40779-020-00257-5 (PMC7288536; doi:10.1186/s40779-020-00257-5)
Supplement: Supplementary file 2 — Additional file 2: Table S2. Correlation analysis of explosion accidents and regional population and economy. [file 40779_2020_257_MOESM2_ESM.docx]

**Supplement Table 2** Correlation analysis of explosion accidents and regional population and economy

| Statistical factor | Regional population/Number of accidents | Regional economy^a^/Number of accidents | National GDP growth rate^b^/Number of accidents |
| --- | --- | --- | --- |
| *r* | 0.470 | -0.372 | 0.629 |
| *P* | 0.008 | 0.040 | 0.028 |

^a^GDP per capita in 2000-2015, ^b^ National GDP growth rate in 2004-2015, *r*: Pearson correlation coefficient.
